# Evaluation and Management of Rotator Cuff Tears Following Shoulder Dislocation

**DOI:** 10.1007/s12178-025-09970-x

**Published:** 2025-05-05

**Authors:** Natalie K. Kucirek, Jamie E. Confino, Brian Feeley, Michael R. Davies

**Affiliations:** UCSF Department of Orthopaedic Surgery, University of California, San Francisco, 1500 Owens Street, San Francisco, CA 94158 USA

**Keywords:** Rotator cuff tear, Shoulder dislocation, Shoulder instability, Rotator cuff repair, Shoulder stabilization

## Abstract

**Purpose of Review:**

This review aims to summarize the epidemiology, associated pathology, and treatment options for rotator cuff tears following shoulder dislocations and to provide a treatment algorithm for these complex injuries.

**Recent Findings:**

Rotator cuff tears after shoulder dislocations most often occur in patients older than 40 and may be more prevalent in women. Up to 50% of these patients may have concomitant Bankart lesions. Patients treated nonoperatively generally have worse functional outcomes and higher pain scores than those who undergo rotator cuff repair. However, further studies are needed to elucidate when the rotator cuff can be repaired in isolation versus in combination with capsulolabral stabilization. Reverse total shoulder arthroplasty is a viable treatment option for elderly patients with irreparable cuff tears after dislocation or post-instability arthropathy.

**Summary:**

Patients who sustain a shoulder dislocation should be evaluated for a rotator cuff tear, particularly if older than 40. Those with post-instability full-thickness rotator cuff tears benefit from surgical treatment. Further research is needed to clarify when additional stabilization procedures should be performed alongside rotator cuff repair.

## Introduction

Glenohumeral dislocations are common orthopaedic injuries, with an estimated incidence of 23.9 per 100,000 person-years in the United States [1]. These dislocations can result in damage to a variety of stabilizing structures of the shoulder joint, including the capsulolabral complex as well as the rotator cuff. Instability-associated rotator cuff tears may be missed and can lead to persistent pain, loss of shoulder function, and recurrent instability if not properly addressed. This review will discuss the epidemiology of rotator cuff tears with glenohumeral instability, highlight various treatment strategies, and propose an algorithm for addressing these injuries.

### Epidemiology of Rotator Cuff Injury After Shoulder Dislocation

Rotator cuff tears are much more commonly seen among older patients after a dislocation event when compared to younger patients. In a study of 240 adult patients who underwent magnetic resonance imaging (MRI) and ultrasound assessment after a traumatic anterior glenohumeral dislocation, 28% of patients were found to have a rotator cuff tear; however, all cuff tears were seen in patients aged 45 years or older [2]. Similarly, in another study of all-comers who sustained an acute primary anterior shoulder dislocation, 63% of patients older than 50 had rotator cuff tears, compared to 0% of patients younger than 50 [3]. McLaughlin hypothesized that in older patients, age-related degeneration of the rotator cuff leads to failure of the dynamic posterior-superior stabilizing structures of the glenohumeral joint during an instability event, whereas in younger patients, the anterior capsulolabral structures are more frequently the point of failure [4].

Rates of rotator cuff tears among older patients who experience shoulder dislocations are variable but substantial, ranging from 35 to 86% [5–10]. As patient age increases, rates of cuff tear after dislocations similarly rise. Among 108 patients older than 60 with an anterior shoulder dislocation, 61% had cuff tears [8]. Another study reported an 83.3% rate of cuff tear in patients who sustained a dislocation between ages 70–80 and 100% in those older than 80 [7]. The retrospective nature of these studies makes it difficult to discern whether cuff tears seen after dislocation events occurred in the setting of the traumatic injury, or whether older patients with asymptomatic, pre-existing cuff pathology were predisposed to having a dislocation event. Berbig et al. compared a cohort patients with primary anterior shoulder dislocations to a group of controls with no prior shoulder dislocations or surgery, finding that among patients > 60 years old, the rate of full-thickness cuff tear in the dislocation group was 56.1%, versus 44.4% among controls [11]. However, the authors reported that 83% of the tears in the dislocation group appeared acute based on ultrasonographic features, which may suggest a component of acute-on-chronic rotator cuff injury in this population.

In contrast, rotator cuff tears are unusual injuries in young patients after shoulder instability events. In a cohort of patients younger than 24 with first-time anterior shoulder dislocations, none of the 63 patients included had sustained a rotator cuff tear based on intraoperative inspection of the cuff [12]. However, one study by Rahu et al. did report that among competitive contact athletes younger than 25 years old who underwent stabilization surgery for first-time dislocation, nine of the sixteen patients were found to have articular-sided partial-thickness rotator cuff tears. All partial tears were treated with debridement alone and there were no reported differences in ASES score at two-year follow up among those with and without them, suggesting minimal functional impact in this young, active population [13].

In addition to older age, female sex may be a risk factor for cuff tear after dislocation. Berbig et al. reported among their cohort, the rate of cuff tear after dislocation in women was 47.7%, compared to 21.6% in men (*p* = 0.0024) [11]. Similarly, in a large study of 3633 patients who suffered traumatic anterior shoulder dislocations, female sex, lower-energy mechanism, and older age were predictors of cuff tear or greater tuberosity fracture [14]. The pathophysiology behind this sex difference is not clear based upon the available evidence and warrants further investigation.

### Injury Patterns and Concomitant Pathology of Rotator Cuff Injury After Shoulder Dislocation

When considering the most common rotator cuff tear patterns that accompany shoulder instability, the supraspinatus tendon is most frequently injured. In a study of first-time traumatic dislocations among 40 patients older than 40 years, 57.5% of patients had full-thickness cuff tears diagnosed on ultrasound, with 96% of these tears involving the supraspinatus [7]. Berbig et al. similarly reported that all 53 cuff tears in their prospective cohort of 167 patients involved the supraspinatus [11]. In a natural history study of patients older than 50 with a first-time shoulder dislocation, 66 patients underwent MRI for persistent pain, of which 62% were found to have a full-thickness cuff tear. Among these tears, 48% were isolated to the supraspinatus, 24% supraspinatus and infraspinatus, 12% combined supraspinatus, infraspinatus, and subscapularis, and 9.6% supraspinatus and subscapularis tears. Only 2.4% had isolated infraspinatus tears and 4.8% had isolated subscapularis tears [6]. Reported tear size also varies in the literature. In a cohort of patients who had sustained dislocations older than age 40, all associated cuff tears were reported to be between 1 and 5 centimeters [5]. Berbig et al. reported 92% of 53 identified tears to be large or massive [11]. In contrast, among a prospectively-collected cohort of 367 patients with cuff tears after dislocation, 37% of tears were greater than 3 centimeters and 48% of tears were between 1 and 3 centimeters [14].

Patients with rotator cuff tears after instability often have accompanying injuries to other stabilizing structures within the shoulder. When intraoperatively assessing injury patterns among patients older than 35 who failed conservative treatment after a traumatic dislocation, one study reported that 64% of patients had a rotator cuff tear, of which 50% had a concomitant Bankart lesion, 28% had a concomitant biceps tendon rupture, 11% had a concomitant humeral avulsion of the glenohumeral ligament (HAGL) lesion, and 100% had a concomitant Hill Sachs lesion [15]. Another study found that among patients older than 40 with cuff tears after first-time shoulder dislocations, 29 of 55 patients (52.7%) had concomitant Bankart lesions. Additionally, the patients with concomitant Bankart lesions were younger than those with isolated cuff tears (61 vs. 69 years, *p* < 0.001) [16]. In a group of all-comers with traumatic anterior dislocations, the most frequent injuries seen alongside cuff tears included Bankart lesions (27%) and axillary nerve injury (22%) [2]. Robinson et al. assessed common injury patterns seen amongst 3633 adult anterior shoulder dislocations, identifying a subset of 7.8% of patients with rotator cuff tears or greater tuberosity fractures after dislocation and concomitant nerve injury. Of this group, 62% of nerve injuries were isolated to the axillary nerve, whereas 22% involved injuries to multiple nerves [14].

Distinguishing nerve injury from cuff pathology can present a challenge. An axillary nerve injury may be incorrectly diagnosed as the cause of inability to abduct after a dislocation, when in fact the patient has a rotator cuff tear [17]. However, surgeons should also be aware of the rare “terrible triad of the shoulder”, in which a patient suffers an anterior shoulder dislocation, rotator cuff tear, and neurologic injury [18]. Another unique dislocation pattern commonly presenting with cuff tear and nerve injury is the luxatio erecta dislocation, in which the humeral head is dislocated inferiorly and the arm remains in an abducted position. Some studies report rates of nerve injury as high as 60% and concomitant cuff tear or greater tuberosity injury in 80% of these patients [19]. Therefore, careful clinical and imaging workup should be done for patients with any range of motion deficits after dislocation.

### Treatment of Rotator Cuff Tears in the Setting of Shoulder Instability

### Operative Versus Nonoperative Management

Treatment options for rotator cuff tears after shoulder dislocations focus on preventing recurrent instability as well as minimizing the pain and loss of function associated with rotator cuff injury. First, it is important to maintain a high index of suspicion for concomitant rotator cuff tears in patients above age 40 who present following a shoulder dislocation. In 1993, Neviaser reported on 37 patients above the age of 40 with undiagnosed rotator cuff tears following a dislocation an average of 7 months prior, who were all unable to forward elevate the affected arm on examination and unable to regain satisfactory function after physical therapy. These findings were mistakenly attributed to axillary nerve palsies by outside physicians, with 11 of the 37 patients experiencing recurrent instability on presentation [10].

In a systematic review of patients diagnosed with rotator cuff tears following shoulder dislocations, patients who were treated nonoperatively had increased pain and decreased functional scores compared to those treated operatively [20]. Of note, several of the included studies did not specify whether tears were full- or partial-thickness. In all four studies comparing nonoperative to operative treatment of rotator cuff tears following shoulder dislocation, patients had improved outcomes after surgery including decreased pain scores, increased function, decreased recurrent instability and higher patient satisfaction than those patients treated nonoperatively [20]. Furthermore, Hawkins et al. found that among 39 older patients treated nonoperatively after an anterior dislocation, the vast majority (90%) had a rotator cuff tear and 30 of 39 (77%) remained symptomatic at an average of 32 months, with two-thirds of these patients reporting persistently impaired motion [21]. In another study of 12 patients over 40 years of age with acute rotator cuff tears following a shoulder dislocation skiing, 84% of patients had excellent or good results when treated surgically with rotator cuff repair, compared to 50% treated non-operatively [9].

However, the decision to perform surgery must be evaluated against the risks of the operation for every patient. A subset of patients with rotator cuff tears after dislocation are elderly and medically fragile and therefore may be poor surgical candidates. When faced with such patients, it is reasonable to treat even large cuff tears with physical therapy and symptomatic management.

### Rotator Cuff Repair Alone

In Simank et al.’s study, patients with rotator cuff tears after dislocation had improved function (*p* = 0.009), decreased pain, and higher satisfaction (*p* = 0.007) after rotator cuff repair alone compared to patients treated nonoperatively [5]. Out of 31 patients treated nonoperatively, 3 patients had recurrent instability (10%) while none of the patients (0%) treated with isolated rotator cuff repair experienced recurrent instability [5]. In another study, 11 patients over the age of 50 with massive rotator cuff tears due to shoulder dislocations underwent arthroscopic rotator cuff repair and achieved satisfactory functional outcomes and range of motion without any recurrence of instability [22].

In a retrospective review of 18 patients who underwent arthroscopic rotator cuff repair after dislocation, on average patients had excellent postoperative patient reported outcomes (VAS 0.6, SSV 89%, ASES 93.1); however, only 53% achieved full strength in external rotation, 71% had full strength in forward flexion, and 88% had full strength on internal rotation testing [23]. There were no complications or reoperations post-operatively. When comparing traumatic rotator cuff tears following shoulder dislocation with traumatic rotator cuff tears without dislocation, the dislocation cohort had significantly lower external rotation strength compared to tears without dislocations [23]. The authors hypothesize that this loss of external rotation strength may have been due to the higher incidence of infraspinatus tearing (78% versus 36%) and larger average tear size seen in their dislocation group (34 mm versus 19 mm) when compared to the no-dislocation group.

In another study of 84 patients over the age of 40 with traumatic rotator cuff tears following anterior shoulder dislocation that underwent isolated arthroscopic rotator cuff repair, only one patient reported recurrent instability (1.2%) at 2.5 years after surgery [24]. Age, presence of a subscapularis tear, presence of a bony Bankart lesion, humeral defects, or neurological injuries were not risk factors for recurrent instability [24]. This suggests that even with a Bankart lesion, isolated rotator cuff repair may be sufficient for restoring stability with low recurrent instability rates. However, ten patients (11.9%) required re-intervention for symptomatic rotator cuff re-tears [24].

In a subset of 23 patients with both a confirmed rotator cuff tear and Bankart lesion following shoulder dislocation that were treated with rotator cuff repair alone, patients had good functional outcome scores, only one patient had a recurrent dislocation (4.3%), and 4 patients (21.1%) had a rotator cuff retear on ultrasound [25]. In summary, the literature suggests that rotator cuff repair alone may be sufficient to restore stability of the shoulder joint after a dislocation-associated cuff tear as rates of recurrent dislocation after isolated cuff repair are low. However, these patients may still be at risk of re-tear of the repaired rotator cuff.

### Rotator Cuff and Labral Repair

The indications for a combined rotator cuff repair with capsulolabral stabilization in the population above age 40 remain poorly defined. While the root cause of instability in this population may be rotator cuff tears as opposed to capsulolabral injury [26], concomitant intra-articular pathology may be increasingly common as patients maintain a higher level of activity later in life. As noted previously, rates of concomitant Bankart lesion and cuff tear after shoulder dislocation have been reported to be between 27 and 53% [2, 15, 16]. Surgeons must therefore decide whether capsulolabral repair, rotator cuff repair, or both should be performed in patients with rotator cuff tears as well as Bankart lesions following shoulder dislocations.

In a retrospective review of 146 patients over the age of 40 comparing anterior shoulder stabilizations with and without rotator cuff repair, there were no statistically significant differences in SANE scores, ASES scores, VAS pain scores or satisfaction stores at 2 year follow up between groups [27]. No patients had recurrence of instability, and Bankart repair with and without rotator cuff repair was effective at restoring function and relieving pain [27]. Additionally, in a retrospective study of 13 patients who underwent arthroscopic combined Bankart and rotator cuff repair following acute shoulder dislocations, there were no significant differences in ASES score, mean Constant score, or mean abduction strength when compared to the contralateral unaffected, non-injured shoulder at a mean of 3 years after surgery [28]. According to ultrasound imaging, there were persistent or recurrent rotator cuff tears in 4/13 patients; however, even in these patients, there were no significant differences in functional outcomes compared to the contralateral shoulder [28].

In a cadaveric study, Shin et al. evaluated supraspinatus tears alone, supraspinatus tears with Bankart lesions, supraspinatus repairs, and supraspinatus repairs combined with Bankart repairs. Rotational range of motion and force required for anteroinferior dislocation were measured at 30 and 60 degrees of abduction. Supraspinatus tears combined with Bankart lesions significantly increased total rotational range of motion as well as decreased the force required for dislocation. Bankart repair combined with supraspinatus repair successfully restored range of motion and increased the force required for dislocation [29].

Although there are promising outcomes following concomitant labral repair and rotator cuff repair in patients with acute rotator cuff tears following shoulder dislocations, there is a lack of literature comparing clinical outcomes of this combined procedure versus rotator cuff repair alone for patients with both Bankart lesions and acute rotator cuff tears. Some surgeons have suggested a combined repair of the rotator cuff tear and Bankart repair in patients under the age of 40; however, there is insufficient evidence to guide this decision [30]. Shin et al. utilize the size of the cuff tear to guide surgical decision making in patients with both a capsulolabral and rotator cuff injury [31]. They cite biomechanical studies in which small cuff tears alone did not compromise overall shoulder stability, whereas larger tears were sufficient to destabilize the shoulder to applied loads [32]. Therefore, in patients with large or massive cuff tears and capsulolabral injuries, the authors repair only the cuff to restore joint stability. In contrast, in patients with small- to medium-sized full-thickness cuff tears, they repair capsulolabral lesions alongside the rotator cuff tears [31].

### Reverse Total Shoulder Arthroplasty

In older patients with significant rotator cuff deficiency after instability events, reverse total shoulder arthroplasty (rTSA) represents an additional viable treatment option. There is limited literature reporting on the use of rTSA specifically in the setting of instability-related rotator cuff injury, though rTSA is commonly used to treat both cuff-tear arthropathy and irreparable rotator cuff tears in older patients. One case report from Shubert et al. described an elderly patient who underwent rTSA after experiencing multiple anterior dislocations beginning in the seventh decade of life in the setting of a massive irreparable rotator cuff tear with poor muscle quality. At two years after surgery, the authors reported no subsequent instability and improved functional outcome measures [33].

rTSA also appears to produce good outcomes in patients who underwent prior shoulder stabilization procedures but had ongoing rotator cuff deficiency and post-instability arthritis, as reported by Raiss et al. in a series of thirteen patients. In this study, 92% of patients were satisfied with their rTSA, there was a significant improvement in Constant scores at an average follow up of 3.5 years, and shoulder flexion improved significantly [34]. An additional study of patients treated with rTSA for dislocation-related arthropathy found that while patients with rotator cuff tears had lower pre- and post-operative Constant scores than those without cuff tears, they achieved better improvements in forward flexion after rTSA [35]. There were also no differences in complication rate or reoperation in those with cuff tears, reinforcing the viability of rTSA for rotator cuff deficiency in the setting of instability. While further studies are needed in this patient population, rTSA appears to be a viable option for patients with irreparable cuff tears or poor muscle quality after dislocation events, or those with repeated instability and cuff deficiency in older age.

### Proposed Treatment Algorithm

**Fig. 1:**
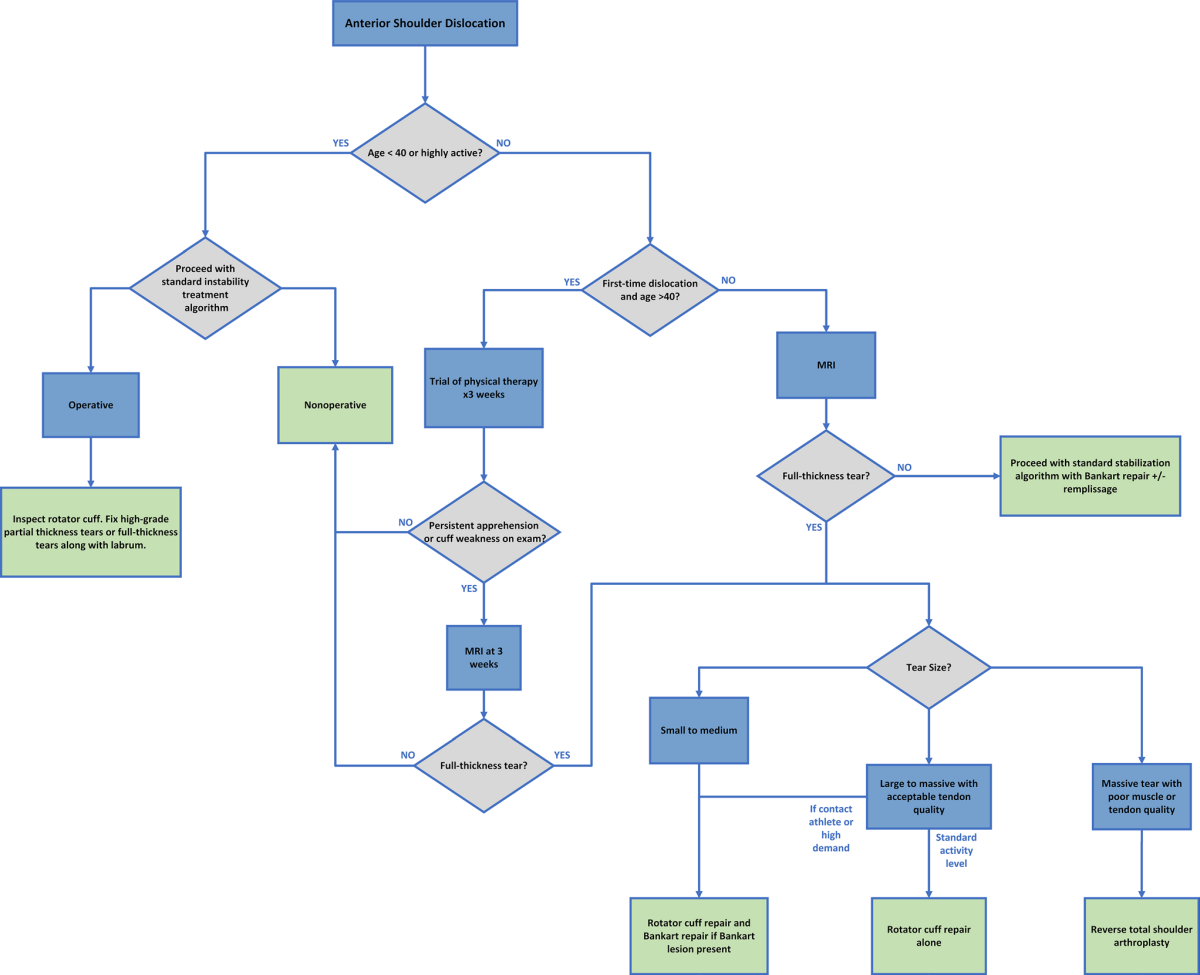
Proposed diagnostic and treatment algorithm for rotator cuff tears after shoulder dislocation

Our proposed treatment algorithm is summarized in Fig. 1 below. For patients younger than forty or with high activity levels who sustain an anterior shoulder dislocation, we recommend following typical shoulder instability guidelines to determine if surgery is indicated. If surgery is indicated, the rotator cuff should be assessed intraoperatively. If there is a high-grade partial-thickness or a full-thickness rotator cuff tear, the tear should be repaired alongside the capsulolabral lesion in the young, active population.

For patients who are roughly age 40 or older presenting with a first-time anterior shoulder dislocation, we recommend a trial of nonoperative management with physical therapy if strength and range of motion are preserved on clinical exam. If these patients have persistent apprehension or weakness of the rotator cuff three weeks after injury, we recommend obtaining an MRI. For older patients with multiple shoulder dislocations or those with persistent weakness at three weeks who are found to have a full-thickness rotator cuff tear on MRI, we recommend operative management. The type of operative intervention will depend on tear size, patient activity level, and rotator cuff tendon and muscle quality.

If an older patient with a nonarthritic glenohumeral joint has repeat dislocations and persistent instability but no rotator cuff tear on MRI, we recommend proceeding down a standard traumatic shoulder instability treatment pathway, likely involving Bankart repair with or without remplissage as clinically indicated, and with an assessment of any glenoid bony deficits to determine if bony augmentation may be necessary. If this patient is found to have a small-to-medium size full-thickness rotator cuff tear in addition to capsulolabral injury, we recommend rotator cuff repair to prevent tear progression along with repair of capsulolabral injuries to restore shoulder stability. If a patient has a large-to-massive rotator cuff tear and has low-to-moderate activity level, we recommend repairing the cuff tear alone even if capsulolabral injuries are present, as we feel that in this case, failure of the posterior-superior cuff is the likely dominant factor causing instability. Lastly, for patients with irreparable cuff tears in the setting of poor muscle or tendon quality and ongoing instability, or those whom already have substantial glenohumeral osteoarthritis, we recommend considering reverse total shoulder arthroplasty.

## Conclusions

Rotator cuff tears in conjunction with shoulder dislocations are commonly seen in patients older than 40, though are rare among younger patients. Increasing age and female sex may increase the risk of rotator cuff tear after dislocation. Cuff tears following instability most commonly affect the supraspinatus tendon but may involve multiple tendons. A subset of patients also experience axillary nerve injury or concomitant shoulder pathology, such as Bankart lesions, biceps tendon injury, and Hill Sachs lesions. Surgical treatment options for rotator cuff tears in this setting include isolated rotator cuff repair, combined rotator cuff and capsulolabral repair, or reverse total shoulder arthroplasty in the setting of irreparable cuff tears or post-dislocation arthropathy. While the optimal surgical treatment algorithm for post-dislocation cuff tears remains controversial, patients sustaining these complex injuries appear to benefit from surgery when compared to nonoperative treatment.

## Key References

- Robinson CM, Shur N, Sharpe T, Ray A, Murray IR. Injuries associated with traumatic anterior glenohumeral dislocations. J Bone Joint Surg Am 2012;94:18–26.
-
  - This large study of over 3000 patients highlights injury patterns seen after shoulder dislocations, provides incidence data on concomitant rotator cuff tears, and identifies older age and female sex as risk factors for rotator cuff tear or greater tuberosity fracture after dislocation.
- Simank H-G, Dauer G, Schneider S, Loew M. Incidence of rotator cuff tears in shoulder dislocations and results of therapy in older patients. Arch Orthop Trauma Surg 2006;126:235–40.
-
  - This study compares nonoperative versus operative treatment of rotator cuff tears sustained after shoulder dislocation in patients older than 40, finding improved outcomes after operative treatment.
- Marsalli M, Errázuriz JDD, Morán NI, Cartaya MA. Recurrence of glenohumeral instability in patients with isolated rotator cuff repair after a traumatic shoulder dislocation. Arch Orthop Trauma Surg 2023;143:3857–62.
-
  - This study reports low rates of recurrent glenohumeral instability for patients treated with isolated rotator cuff repair after shoulder dislocation, suggesting that cuff repair alone may be sufficient to restore shoulder stability in many patients.
- Eibel A, Reddy RP, Hughes JD, Smith C, Popchak A, West R, et al. Traumatic rotator cuff tears with concomitant shoulder dislocation: tear characteristics and postsurgical outcomes. Journal of Shoulder and Elbow Surgery 2023;32:842–9.
-
  - This study compares patients undergoing repair of rotator cuff tears sustained in the presence or absence of a shoulder dislocation, finding that post-instability rotator cuff tears were larger but that patients in both groups had similarly improved patient-reported outcomes.

## Data Availability

No datasets were generated or analysed during the current study.
